# Primary presentation and clinical course of pediatric and adolescent patients with differentiated thyroid carcinoma after radioiodine therapy

**DOI:** 10.3389/fonc.2023.1237472

**Published:** 2023-10-02

**Authors:** Caroline Burgard, Mathias Johannes Zacherl, Andrei Todica, Julia Hornung, Freba Grawe, Isabell Pekrul, Petra Zimmermann, Christine Schmid-Tannwald, Roland Ladurner, Detlef Krenz, Arnold Trupka, Johanna Wagner, Peter Bartenstein, Christine Spitzweg, Vera Wenter

**Affiliations:** ^1^ Department of Nuclear Medicine, LMU University Hospital, LMU Munich, Munich, Germany; ^2^ Department of Nuclear Medicine, Saarland University, UdS, Homburg, Germany; ^3^ Interdisciplinary Center for Thyroid Carcinoma (ISKUM), LMU University Hospital, LMU Munich, Munich, Germany; ^4^ Department of Radiology, LMU University Hospital, LMU Munich, Munich, Germany; ^5^ Department of Anaesthesiology, LMU University Hospital, LMU Munich, Munich, Germany; ^6^ Department of Visceral and Endocrinological Surgery, LMU University Hospital, LMU Munich, Munich, Germany; ^7^ Department of Surgery, Martha-Maria Krankenhaus, Munich, Germany; ^8^ Department of Surgery, Klinikum Dritter Orden, Munich, Germany; ^9^ Department of Endocrine Surgery, Starnberg Hospital, Starnberg, Germany; ^10^ Department of Pediatric Neurology, Developmental Medicine and Social Pediatrics, Dr. von Hauner Children’s Hospital, LMU University Hospital, LMU Munich, Munich, Germany; ^11^ Department of Internal Medicine IV, LMU University Hospital, LMU Munich, Munich, Germany

**Keywords:** thyroid carcinoma, radioiodine therapy, pediatric oncology, children, adolescents, patient outcome

## Abstract

**Introduction:**

Differentiated thyroid carcinoma (DTC) in childhood and during adolescence is extremely rare. Pediatric DTC commonly presents with advanced disease at diagnosis including a high prevalence of cervical lymph node metastases and pulmonary metastases. Studies in children with DTC are limited. Therefore, we aimed to evaluate the initial presentation, effectiveness of radioiodine therapy (RIT), and long-term outcome of prepubertal in comparison to pubertal/postpubertal patients.

**Methods:**

Eighty-five pediatric and young patients aged 6.4 to 21.9 years with histopathologically confirmed DTC were retrospectively included. They all underwent total thyroidectomy followed by RIT. Initial presentation and outcome of prepubertal and pubertal/postpubertal patients were compared 1 year after RIT, during follow-up, and at the last visit of follow-up.

**Results:**

Prepubertal patients presented with significantly higher T and M stages. One year after RIT, 42/81 (52%) patients still presented with evidence of disease (ED). During follow-up of a median of 7.9 years, prepubertal patients were less often in complete remission (58% vs. 82% in pubertal patients). At the last visit of follow-up, 19/80 (24%) patients still had ED without statistical differences between the two groups (42% prepubertal vs. 18% pubertal/postpubertal, *p*-value 0.06). None of our patients died disease-related over the observed period.

**Conclusion:**

Prepubertal children with DTC presented with a more advanced tumor stage at the initial presentation. During follow-up, they present more often with ED. However, at the end of our study, we did not observe statistically relevant differences in patient outcomes between the prepubertal and pubertal/postpubertal groups.

## 1 Introduction

Differentiated thyroid carcinoma (DTC) is the most common endocrine tumor in adults. In childhood and during adolescence, however, thyroid carcinomas are extremely rare and account for <2% of all diagnosed thyroid carcinoma cases (1). However, its worldwide incidence is increasing (2). Papillary thyroid carcinoma (PTC) accounts for 90% or more of all cases diagnosed in childhood, while follicular thyroid carcinoma (FTC) is rarely diagnosed (3, 4).

Initial presentation and clinical course appear to be different from adults (5). RET-PTC and NTRK fusions present the most common genetic alterations, while mutations in BRAF V600E and RAS point mutations are less frequently seen (6, 7). Children often present with a more aggressive and advanced stage of disease at initial diagnosis, i.e., locoregional lymph node metastases in 39%–90% of the cases and distant metastases in 6%–41% of the cases (1, 8) presenting most often as pulmonary metastases and more frequently in a disseminated manner compared with adults (9). In addition, thyroid cancer in childhood is associated with a higher recurrence rate (11%–45%) (1). Nevertheless, the prognosis of pediatric thyroid cancer is more favorable than in adults with less than 5% of fatal outcomes (4, 10).

The current standard treatment for most pediatric thyroid cancer patients is surgery (4). After total thyroidectomy (TE) for histologically confirmed DTC, the recommendations for the application of radioiodine vary and have changed in the last decade (4, 11–13). Most recently, the European Thyroid Association (ETA) has published new guidelines for the management of pediatric DTC (13). The use of radioiodine therapy (RIT) is not clearly defined, with uncertainty regarding which patients should receive it and the intended objective (remnant ablation, adjuvant treatment, or treatment of existing disease) (13). Currently, RIT is recommended after TE to treat persistent locoregional disease, remnant thyroid cells, untreatable nodal disease, and iodine avid distant disease (13). Unfortunately, studies in children with DTC treated by TE and followed by [^131^I] NaI therapy are still limited and most often of low evidence level. Therefore, we aimed to evaluate the initial presentation, effectiveness of RIT, and long-term outcome of a pediatric patient cohort with DTC at our center.

## 2 Materials and methods

### 2.1 Patients

We retrospectively reviewed 85 pediatric and adolescent patients, aged between 6.4 and 21.9 years with histopathologically confirmed DTC who underwent initial RIT at our department from 1993 to 2020. The included age was similar to that of previously published studies (14–20). Epidemiological and clinical features of these patients were assessed (age at diagnosis, gender, concomitant disease, TNM stage, tumor size, presence of extrathyroidal extension, resection margins, as well as long-term complications of the operation). TNM staging was based on the AJCC 8th edition in all patients (21). In analogy to previous studies, prepubertal and pubertal/postpubertal children were analyzed separately (22, 23). During follow-up visits, the patient’s physical development was documented. In female patients, the beginning of puberty was defined by the onset of thelarche, which indicates entry into Tanner stage 2. On the other hand, in male patients, Tanner stage 2 was defined by the occurrence of gonadarche, marked by the initial increase in testicular volume. If physiological changes were not sufficiently documented, the cut of age for the onset of puberty was 14 years based on a previously published study (24).

### 2.2 Treatment

All patients underwent total or near-total TE with or without lymphadenectomy (LAE) and were referred to our clinic for RIT. Before RIT, an ultrasound examination of the thyroid bed and the cervical lymph node levels I–VI was performed to exclude large thyroid remnants or cervical lymph node metastases. Twenty-five to 50 MBq [^131^I] NaI (0.7–1.4 mCi) per kg body weight for remnant ablation and adjuvant treatment and 100–150 MBq [^131^I] NaI (2.7–4.1 mCi) per kg body weight for therapy of known metastases were applied which is compatible to current guideline recommendations for children (25). Patients ≥18 years of age were treated with standardized activities of 2,000 (54 mCi), 3,700 (100 mCi), 7,400 (200 mCi) MBq [^131^I] NaI. From 1993 to 2013, patients presenting with any T, any N, any R, and M0 were treated with 3,700 MBq [^131^I] NaI. Additionally, 7,400 MBq [^131^I] NaI was administered if patients presented with M1 stage or if patients had multiple risk factors including advanced T (multifocal T3/T4 tumors with gross extrathyroidal extension), extensive regional lymph node metastases, and R1 stage contemporaneously. After 2013, patients were treated with either 2,000 MBq (any T, any N, M0, R0), 3,700 MBq (any T, any N, M0, R1, or any T, advanced N1, M0, R0), or 7,400 MBq [^131^I] NaI (likewise before 2013). RIT was performed either after thyroid hormone withdrawal [thyroid-stimulating hormone (TSH) ≥30 μU/mL] (*N* = 54) or after application of recombinant human thyrotropin alfa (rhTSH, Thyrogen^®^, Sanofi Genzyme, Cambridge, MA, United States) on two consecutive days (*N* = 31). The indication of rhTSH has been evaluated on an individual basis. Planar whole-body scans (WBS) and single photon emission computed tomography (SPECT) with or without low-dose CT of the neck and thorax were obtained 72 h after oral administration of [^131^I] NaI using either the Siemens Symbia T, the Symbia Intevo, or the e.cam (all Siemens Healthcare GmbH, 91052 Erlangen, Germany) dual-head multifunctional gamma camera with high-energy high-resolution all-purpose collimator. During follow-up, in low-risk patients (disease grossly confined to the thyroid with N0/Nx disease), TSH was normalized to the lower normal range of 0.3–1.0 mIU/L; in intermediate-risk patients (N1a or minimal N1b disease), the TSH goal was 0.1–0.5 mIU/L; and in high-risk patients (regionally extensive disease, distant metastasis) and in case of evidence of disease (ED), TSH was completely suppressed (<0.1 mIU/L) in adaption to ATA guidelines for children (4).

### 2.3 Outcome

Patient outcome was analyzed at three different time points: 1 year after RIT, during follow-up, and at the last visit of follow-up. During the first year after RIT, follow-up examinations [including physical examination, ultrasound of the thyroid bed and cervical lymph node compartments, determination of thyroglobulin (Tg), Tg antibodies, Tg recovery, TSH, fT3, fT4, and routine laboratory] were performed every 3 months. After 3–12 months, a diagnostic WBS with 370 MBq [^131^I] NaI (10 mCi) was performed in hypothyroidism if further cycles of RIT were not indicated. An additional SPECT/(low-dose CT) was performed (most often of the neck and thorax region) if pathological uptake was present in the planar WBS. No evidence of disease (NED) was assumed if WBS, SPECT/(CT), and ultrasound were unremarkable and if Tg was under the detection limit in the absence of Tg antibodies and with undisturbed Tg recovery. In contrast, ED was expected if pathological findings were diagnosed in imaging studies and/or if Tg was measurable or if Tg antibodies were significantly increased. On outcome during follow-up, during the second and third year of follow-up, follow-up examinations were performed every 6 months and annually thereafter in patients with NED. In patients with ED, follow-up examinations were planned individually. In patients with NED 1 year after RIT (*N* = 39), the recurrence rate was evaluated. Recurrence was supposed if the Tg level rose above the detection limit or in case of pathological imaging findings. Furthermore, during follow-up, patients were categorized into complete remission, partial remission, stable disease, and progressive disease. These categories were defined as described before (26). Additionally, at the last visit of follow-up, patient outcome was categorized as ED/NED (no clinical findings in ultrasound examination, no biochemical ED with basal serum Tg <0.1, negative Tg antibodies), indeterminate response (a mild elevation of Tg, positive anti-Tg antibodies cannot exclude residual or recurrent disease and/or non-specific imaging findings), biochemical incomplete response (significant elevation of serum Tg >1 ng/mL in the absence of structural ED), and structural incomplete response (presence of disease in imaging accompanied by a significant elevation of serum Tg or positive Tg antibodies) as described previously (27). In addition, survival was evaluated. To assess psycho-oncological distress during follow-up, the German version of the National Comprehensive Cancer Network (NCCN) Distress-Thermometer was used. It consists of a scale ranging from 0 to 10 (28).

### 2.4 Statistical analysis

Statistical analyses were performed using the SPSS software package (IBM SPSS Statistics 27.0). A *p*-value of less than 0.05 was assumed to be statistically significant. Comparisons of variables between the prepubertal group and the pubertal/postpubertal group were performed using Student’s *t*-test, chi-square test, or Mann–Whitney *U*-test. Continuous variables were reported as mean ± standard deviation (SD). Categorical variables were reported as numbers and percentages.

## 3 Results

### 3.1 Patients

**Table 1** summarizes the patient baseline characteristics at the primary presentation.

**Table 1:** Baseline characteristics at primary presentation.

Characteristics	Total (%)	Prepubertal	Pubertal/postpubertal	*p*-value
	*N* = 85	*N* = 20 (24%)	*N* = 65 (76%)	
Male Female	18 (21%) 67 (79%)	6 (30%) 14 (70%)	12 (19%) 53 (82%)	0.348
Age at primary surgery (years)	17.01 ± 3.5 range (6.4–21.9)	12.2 ± 2.2 range (6.4–14.3)	18.5 ± 2.3 (14.0–21.9)	<0.001
TE TE and LAE	8 (9%) 77 (91%)	1 (5%) 19 (95%)	7 (11%) 58 (89%)	0.674
Tumor diameter (cm)	2.2 ± 1.3	2.7 ± 1.3	2.1 ± 1.3	0.072
Extrathyroidal extension	35/85 (41%)	11/20 (55%)	24/65 (37%)	0.196
Multifocality	25 (30%)	6 (30%)	19 (29%)	1.0
Mean initial RAI dose in MBq (mCi)	3,584 ± 1,254 (96 ± 34)	3,110 ± 1,507 (84 ± 40)	3,730 ± 1,140 (100 ± 30)	0.053
Mean cumulative RAI dose in MBq (mCi)	7,477 ± 6,709 (197 ± 179)	6,517 ± 5,755 (161 ± 151)	7,772 ± 6,990 (210 ± 188)	0.246
Median time between surgery and RIT (days)	36 (14; 177)	32 (14; 77)	38 (16; 177)	0.045
Median time of follow-up (years)	7.9 (1.1; 26.1)	7.5 (1.6; 22.1)	8.0 (1.1; 26.1)	0.189

TE, total thyroidectomy; LAE, lymphadenopathy; RIT, radioiodine therapy; RAI, radioactive iodine; MBq, megabecquerel; TSH, thyroid-stimulating hormone; Tg, thyroglobulin; IU, international unit.

Eighty-five patients [67 female (79%) and 18 male (21%) patients; mean age at operation 17.01 ± 3.5 years; min. 6.4 years; max. 21.9 years] were included. Seventy-seven patients (91%) were treated with TE and lymphadenectomy (LAE), and eight patients (9%) underwent TE only. The majority of patients presented with papillary carcinoma (**Table 2**).

**Table 2:** Tumor histology.

Tumor histology	Total (%)	Prepubertal	Pubertal/postpubertal	*p*-value
	*N* = 85	*N* = 20 (24%)	*N* = 65 (76%)	
Papillary carcinoma (all variants)	79 (93%)	19 (95%)	60 (92%)	1.0
• Classical variant	58	10	48	
• Follicular variant	16	7	9	
• Sclerosing variant	3	1	2	
• Tall cell variant	1	0	1	
• Oncocytic variant	1	1	0	
Follicular carcinoma	6 (7%)	1 (5%)	5 (6%)	

**Figure 1:**
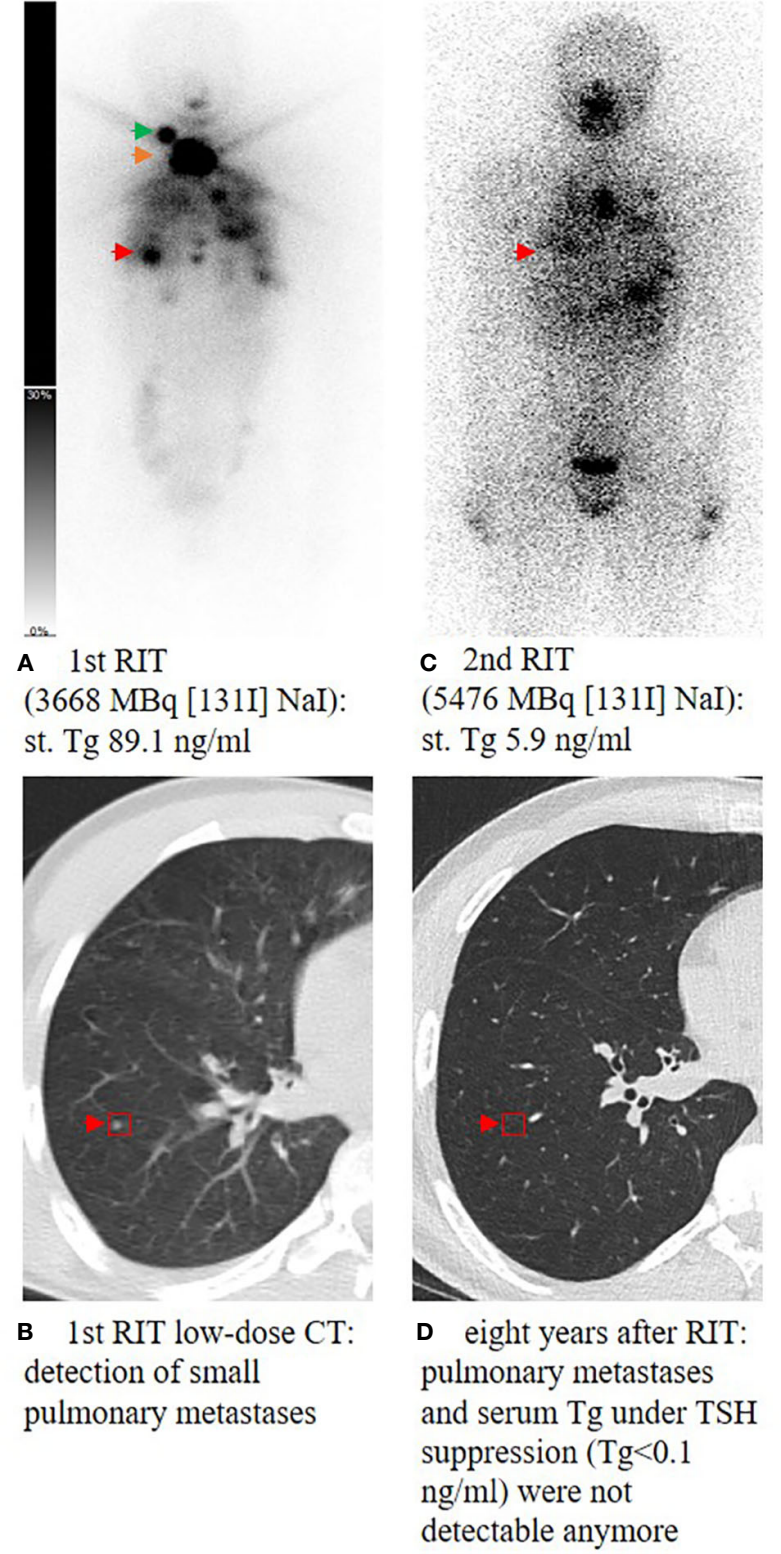
Planar whole-body scintigraphy and CT scan after the first radioiodine therapy (RIT) and during follow-up. A 14-year-old male patient **(A)** presenting with high residual thyroid bed uptake (orange arrow), an iodine-positive cervical lymph node metastasis in level II on the right side (green arrow), and multiple iodine-positive pulmonary metastases (red arrow) **(B)**. Post-therapeutic scan of the second RIT showed ablation of the remnant thyroid tissue and significant uptake of pulmonary metastases **(C)**. During follow-up, TSH was suppressed consequently; 1.6 years after the last RIT, Tg decreased to <0.1 ng/mL and pulmonary metastases could not be detected anymore in the subsequent CT scans **(D)**. At the last visit, 8 years after the first RIT, the patient still presented with excellent response with Tg <0.1 ng/mL and no remarkable findings in computer tomography of the lung. st., stimulated; Tg, thyroglobulin; RIT, radioiodine therapy; TSH, thyroid-stimulating hormone.

At the time of surgery, 20 patients were prepubertal and 65 patients were pubertal/postpubertal. Tumor-node-metastasis (TNM) classification is presented in **Table 3**. Notably, prepubertal patients presented with significantly higher T and M stages. Furthermore, prepubertal patients presented more often with extrathyroidal extension and were diagnosed more often with R1 and N1 stage without reaching statistical significance (*p*-value 0.196, 0.109, and 0.102, respectively). RIT with an initial dose of 3,584 ± 1,254 MBq [^131^I] NaI (96 ± 34 mCi) was performed within a median of 36 days (14; 177 days) after surgery. At the time of the first RIT, distant metastases were unknown. The first RIT was performed with the aim of either remnant ablation in four prepubertal patients (20%) or adjuvant therapy in 16 prepubertal patients (80%) and either remnant ablation in 19 pubertal/postpubertal patients (29%) or adjuvant therapy in 46 pubertal/postpubertal patients (71%; *p* = 0.568). In the post-therapy [^131^I] NaI scan, cervical lymph node metastases were detected in 8/85 patients (4/20 prepubertal; 4/65 pubertal/postpubertal). Radioiodine-positive distant metastases were diagnosed in six patients (7%; 2/20 prepubertal; 4/65 pubertal/postpubertal; **Figure 1**).

**Table 3:** TNM classification.

TNM classification	Total (%)	Prepubertal	Pubertal/postpubertal	*p*-value
	*N* = 85	*N* = 20 (24%)	*N* = 65 (76%)	
pT1/2 pT3/4 pTx	47 (55%) 36 (43%) 2 (2%)	7 (35%) 13 (65%) 0	40 (62%) 23 (35%) 2 (3%)	0.038
pN0/x pN1	30 (35%) 55 (65%)	4 (20%) 16 (80%)	26 (40%) 39 (60%)	0.102
cM0/x cM1	76 (89%) 9 (11%)	15 (75%) 5 (25%)	61 (94%) 4 (6%)	0.03
pR0 pRx/1	56 (66%) 29 (34%)	10 (50%) 10 (50%)	46 (71%) 19 (29%)	0.109

T, tumor; N, nodus; M, metastasis; R, resection; p, histopathological; c, clinical; x, unknown value.

### 3.2 Concomitant disease and other diagnosis

Three patients presented with congenital diseases: one patient with mosaic trisomy 21 and congenial immune defect, one patient with hereditary spherocytosis, and one patient with Morbus Meulengracht. Other diseases such as generalized idiopathic epilepsy, paraganglioma, and scoliosis were documented in three patients, also prior to RIT.

After RIT during follow-up, other malignant diseases were diagnosed in two patients. An indolent non-Hodgkin lymphoma/marginal zone lymphoma of the lungs was diagnosed in the patient with mosaic trisomy 21 and congenial immune defect 11 years after diagnosis of DTC and RIT. Another patient suffered from vulva carcinoma 11 years after the initial diagnosis of DTC.

Furthermore, autoimmune-related diseases were diagnosed during follow-up in five patients [allergic rhinitis/asthma (*N* = 2), neurodermatitis, autoimmune gastritis, and autoimmune hair loss]. One patient reported subclinical hyperandrogenemia. Finally, one patient with ventricle septum defect suffered from stroke 2 years after diagnosis of DTC.

### 3.3 Postoperative complications

Forty-two patients (49%; 9 prepubertal, 33 pubertal/postpubertal) suffered from one or more transient or persistent postoperative complications. Transient hypocalcemia due to parathyroid gland dysfunction presented as the most common postoperative complication in 19 patients [22%; 3/20 prepubertal (15%); 16/65 pubertal/postpubertal (25%)]. Permanent hypoparathyroidism was found in 12 patients [14%; 4/20 prepubertal (20%); 8/65 pubertal/postpubertal (12%)]. Transient vocal cord paralysis on one side due to recurrent laryngeal nerve injury was seen in eight patients [9%; 2/20 prepubertal (10%); 6/65 pubertal/postpubertal (9%)]. One patient in each group suffered from permanent vocal cord palsy. Two patients (10%) in the prepubertal group had to be intubated and ventilated in the intensive care unit due to postoperative respiratory insufficiency. Three patients [4%; 1/20 prepubertal (5%); 2/65 pubertal/postpubertal (3%)] suffered from transient postoperative Horner syndrome. Postoperative bleeding occurred in two prepubertal patients (3%) and wound infection in one postpubertal patient (1%), respectively.

### 3.4 Outcome analysis

**Figure 2:**
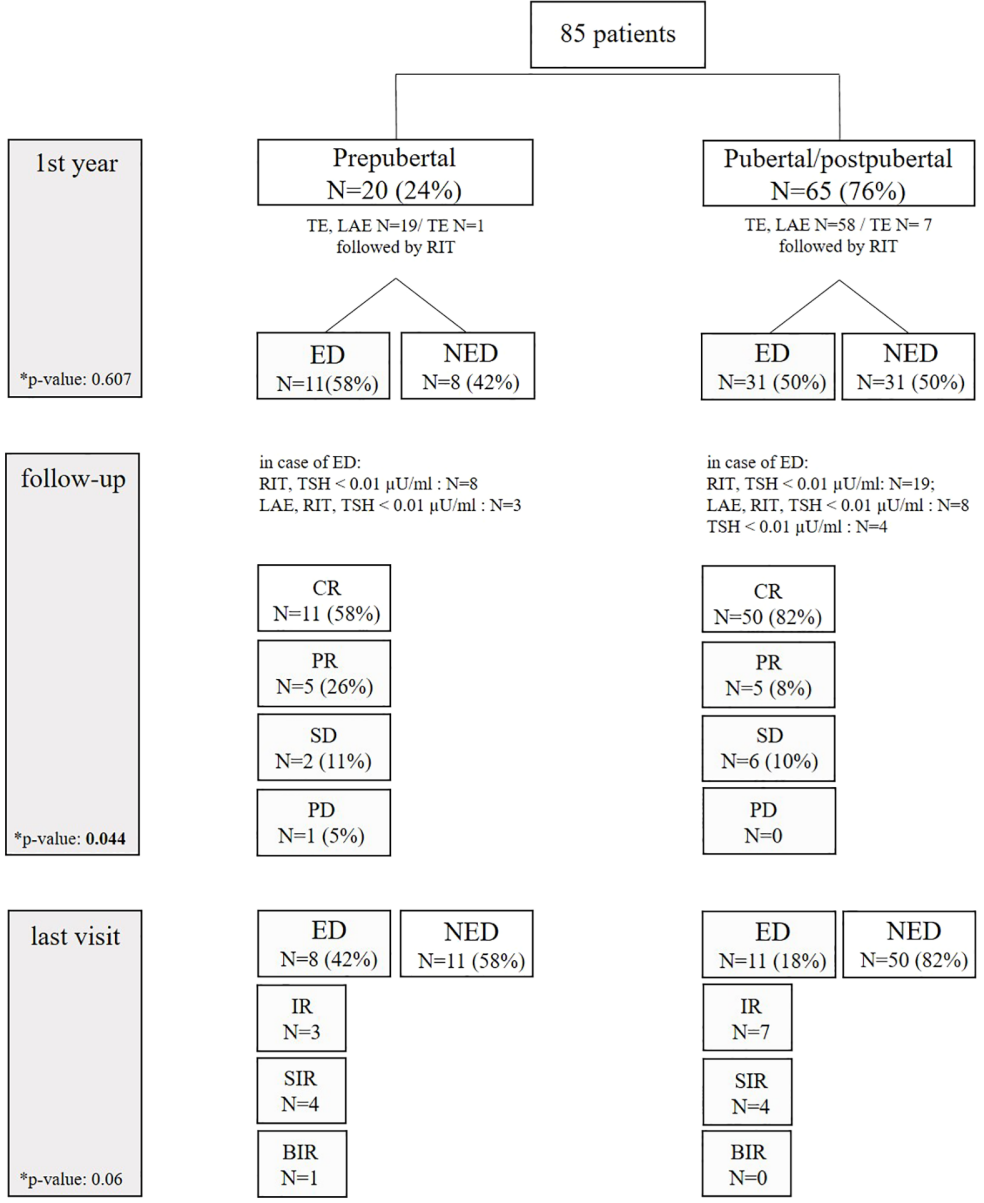
Clinical course of prepubertal and pubertal/postpubertal patients. ED, evidence of disease; NED, no evidence of disease; TE, thyroidectomy; LAE, lymphadenectomy; RIT, radioiodine therapy; CR, complete remission; PR, partial remission; SD, stable disease; PD, progressive disease; IR, indeterminate response; SIR, structural incomplete response; BIR, biochemical incomplete response.

An overview of the outcome in pre- and postpubertal patients with differentiated thyroid carcinoma can be found in **Table 4** and in **Figure 2**.

**Table 4:** Outcome of all patients with differentiated thyroid carcinoma.

	Total (%)	Prepubertal	Pubertal/postpubertal	*p*-value
	*N* = 85	*N* = 20 (24%)	*N* = 65 (76%)	
**Pathological findings in I-131 post-therapeutic scan**	14 (20%)	6 (30%)	8 (12%)	0.069
• Iodine uptake in lymph node metastases:	8	4	4	
• Iodine uptake in distant metastases:	6 (*N* = 5 pulmonary, *N* = 1 bone)	2 (*N* = 2 pulmonary)	4 (*N* = 3 pulmonary; *N* = 1 bone)	
• Non-iodine avid metastases:	2 (*N* = 2 pulmonary)	2 (*N* = 2 pulmonary)	0	
Outcome after 1 year				
• Not available	4	1	3	
• NED	39 (48%)	8 (42%)	31 (50%)	0.607
• ED	42 (52%)	11 (58%)	31 (50%)	
Follow-up				
• Not available	5	1	4	
• Complete remission	61 (76%)	11 (58%)	50 (82%)	0.044
• Partial remission	10 (13%)	5 (26%)	5 (8%)	
• Stable disease	8 (10%)	2 (11%)	6 (10%)	
• Progressive disease • Recurrence	1 (1%) 0/39	1 (5%) 0/8	0 0/31	
Last visit of follow-up				
• Not available	5	1	4	
• NED/ER	61 (76%)	11 (58%)	50 (82%)	0.06
• ED	19 (24%)	8 (42%)	11 (18%)	
o Indeterminate response	10	3	7	0.105
o Biochemical incomplete response	1	1	0	
o Structural incomplete response	8	4	4	
End of the study				
• Not available	5	1	4	
• Survivors	79	19	60	0.838
• Death	1 (not cancer-related)	0	1	

ED, evidence of disease; NED, no evidence of disease; ER, excellent response.

One year after initial diagnosis, 39 patients (48%) presented with NED (08/20 prepubertal; 31/62 pubertal/postpubertal). Vice versa, 42 patients (52%) still had ED (4 patients, loss of follow-up). Two prepubertal patients had radioiodine refractory pulmonary metastases. During follow-up, patients with ED were treated with lymphadenectomy followed by RIT (*N* = 11), further cycles of RIT (*N* = 27), or TSH suppression solely (*N* = 4). In the course of follow-up, 61 patients (76%) were in complete remission (58% of prepubertal vs. 82% of pubertal/postpuberal patients). Partial remission was achieved in 13% (26% in prepubertal vs. 8% in pubertal/postpubertal), stable disease in 10% (11% in prepubertal vs. 10% in pubertal/postpubertal), and progression in 1% (1% in prepubertal vs. 0% in pubertal/postpubertal. These differences were statistically significant (p-value 0.044). Of note, recurrent disease in the subgroup of 39 patients who were disease-free in the first control was not observed during further follow-up.

At the end of the study, ED was observed more often in prepubertal patients (42% prepubertal vs. 18% pubertal/postpubertal), without being statistically different (*p*-value 0.06). In detail, structural incomplete response was seen in eight patients: four prepubertal patients presented with pulmonary metastases (*N* = 2), cervical metastases (*N* = 1), and both (*N* = 1). Four pubertal/postpubertal patients had pulmonary metastases (*N* = 1), cervical metastases (*N* = 2), and both (*N* = 1). Prepubertal/postpubertal patients with indeterminate response had Tg antibodies ranging from 527 to 1,325 IU/mL or Tg ranging from 0.4 to 0.8 ng/mL. Pubertal patients with indeterminate response had Tg antibodies ranging from 46 to 110 IU/mL or a slightly elevated Tg of 0.5 ng/mL. Biochemical incomplete response was seen in one prepubertal patient with a Tg of 1.4 ng/mL in the absence of structural ED.

One patient died due to a stroke unrelated to thyroid cancer. None of our patients died disease-related.

Psycho-oncological distress score was available in 49 patients. The score did not significantly differ in both groups: 3/10 (0/10–6/10) in prepubertal and 4/10 (0/10–8/10) in pubertal/postpubertal patients (*p*-value 0.447). A distress score ≥5 was diagnosed in 25 patients (40% of prepubertal patients vs. 54% of pubertal/postpubertal patients, *p*-value 0.496).

## 4 Discussion

Thyroid cancer is rare in children and adolescents and characteristically shows a different clinical presentation and course of disease as compared with adults (9). Children present more often with aggressive, advanced tumor stage including extensive regional lymph node metastases and distant lung metastases (5, 8, 22, 23, 29–31). These findings are in line with the significantly higher number of prepubertal patients presenting cM1 stage (25%) in our study. In addition, prepubertal patients tended to be diagnosed more often with extrathyroidal extension. Indeed, the small size of the thyroids in younger patients enables rapid tumor to spread beyond the thyroid capsule and invasion of adjacent tissues (22).

The optimal management of DTC in children and adolescents is still controversial. In our retrospective analysis, all patients were treated with total TE. In an earlier study, pediatric patients treated with bilateral lobar resection had a significantly lower risk of local and regional recurrence when compared with pediatric patients treated with hemithyroidectomy (32). However, in the debate on the optimal surgical approach, the risk of postoperative complications must be weighed against the probability of persistent or recurrent disease. In our study, 12 patients suffered from permanent hypoparathyroidism, and one patient from each group suffered from permanent vocal cord palsy. Near-total TE may be an alternative to total TE to minimize the risk of these complications. In a previously published study, including 52 patients ≤15 years and 175 patients >15 years (mean age 18 years), total TE in comparison to less than TE was not associated with disease-free survival in multivariate analysis (33). However, the outcome of prepubertal pediatric DTC after total TE in comparison to near-total TE is still insufficiently investigated. Therefore, prospective studies are needed to evaluate the impact of limited surgery. Hitherto, ATA and ETA guidelines recommend total TE for the majority of children in the hands of very experienced pediatric thyroid surgeons (4, 13). Incidentally found, very small DTC with non-aggressive histological features is excluded and hemithyroidectomy may be considered (13). In our study, the majority of prepubertal children presented with locally advanced tumor growth and cervical lymph node metastases which has guided the surgical approach of TE as a prerequisite for RIT. Therefore, we suggest total TE in children which should be performed by very experienced thyroid surgeons with skills in pediatric surgery. To minimize complications, some millimeters of the thyroid capsule at the vascular pedicle of the parathyroid can be preserved to maintain perfusion of the parathyroid.

All of our patients received RIT either for remnant ablation or for adjuvant treatment and treatment of known disease in accordance with national guidelines (11). Indeed, RIT has been demonstrated to be extremely beneficial, even in children with advanced disseminated pulmonary metastases (13). Pawelczak et al. systematically reviewed the published literature on children and adolescents with metastatic PTC of the lung who were treated by RIT and reported an average complete and partial response rate of 47.3% and 38.4%, respectively (34). In recent years, the distinction of radioiodine application for remnant ablation and for adjuvant treatment has been established which was not the case at the time of initial inclusion of our patients. In our study, RIT was performed primarily for ablation purposes in one of four patients. So far, it has not been clarified if adjuvant RIT can improve survival or reduce recurrence in children (13). The potential late toxicity of RIT in children with their long life expectancy must be carefully taken into consideration. Pediatric and young patients <25 years old who receive RIT for DTC experience a small but increased risk of secondary malignancy, mainly salivary gland cancer. The children’s risk appears to be marginally higher than in adult patients (35) which is of high clinical impact as children with their long life expectancy have a risk of experiencing side effects.

During follow-up, we observed that prepubertal patients were less often in complete remission. However, at the end of the study, we did not find a statistical difference in outcome between the two groups. The outcome of DTC in young children remains a controversial issue. DTC in patients <10 years seems to be associated with higher recurrence and mortality rates in comparison to DTC in older children (36, 37). In contrast, similar clinical courses of DTC in younger children and adolescents have been described (22, 23, 38, 39). Recently, Redlich et al. have confirmed young age and the ATA high-risk group as significant negative prognostic risk factors for event-free survival (40). Overall, 30-year survival rates of 90%–99% are observed in children with DTC, even in the presence of metastatic disease (41–44). None of our patients died disease-related over the observed period.

In our analysis, high-risk patients and patients with ED during follow-up were treated with complete TSH suppression. TSH suppressive therapy in children has been shown to be associated with diastolic dysfunction (45). Furthermore, in patients with hypoparathyroidism, TSH suppression accounts for loss of bone mineral density (45, 46). We have observed that TSH suppression was beneficial in patients with ED leading to a decrease in Tg even under the detection limit. Thus, our data suggest that pediatric patients with ED benefit from TSH suppression. Nevertheless, TSH suppression should be carried out with caution and only transiently taking into consideration the comorbidities.

Our study contains several limitations, including its retrospective and monocentric design. The histopathological data had to be adapted to the eighth edition of the UICC to compare the TNM status. As a result, TNM classification could not be adapted in some patients. Third, SPECT or even hybrid imaging was not available at the time of inclusion in some patients, so that reliable differentiation between residual thyroid tissue and radioiodine avid lymph node metastases was not possible in some cases. Finally, the median follow-up time of nearly 8 years may be too short to detect late recurrent disease and to document overall survival. The good overall prognosis of differentiated thyroid cancer in children and young patients requires a larger cohort of patients and an extremely long follow-up period to demonstrate differences in survival.

## 5 Conclusion

In conclusion, prepubertal children with DTC presented with a more advanced tumor stage at the initial presentation. During follow-up and at the end of our study, we did not observe statistically relevant differences in patient outcomes.

## Data availability statement

The raw data supporting the conclusions of this article will be made available by the authors, without undue reservation.

## Ethics statement

The studies involving humans were approved by the Ethics committee of the Medical Faculty, LMU University Hospital, LMU Munich, Munich, Germany, IRB # 21-0206. The studies were conducted in accordance with the local legislation and institutional requirements. Written informed consent for participation was not required from the participants or the participants’ legal guardians/next of kin because the requirement to obtain informed consent was waived due to the retrospective design of this study. Written informed consent was obtained from the minor(s)’ legal guardian/next of kin for the publication of any potentially identifiable images or data included in this article.

## Author contributions

All authors contributed to the study conception and design. Conceptualization: CB, ATo, and VW. Data curation: CB, ATo, and VW. Formal analysis: CB, MZ, FG, and VW. Investigation: CB, MZ, JH, PZ, RL, DK, and ATr. Methodology: ATo, FG, and VW. Project administration: CB, ATo, PB, and VW. Software: CB and MZ. Supervision: JH, IP, PZ, CS-T, RL, DK, ATr, JW, PB, and CS. Validation: JH. Visualization: MZ. Writing—original draft: CB and VW. Writing—review and editing: CB and VW. All authors commented on previous versions of the manuscript and read and approved the final manuscript.
